# *Peyerimhoffia jaschhoforum* (Diptera, Sciaridae), a new deadwood inhabiting species from Canada

**DOI:** 10.3897/BDJ.2.e4200

**Published:** 2014-11-17

**Authors:** Rob Deady, Kai Heller, Timothy Work, Lisa Venier

**Affiliations:** † Université du Québec à Montréal (UQÀM), Montréal, Canada; ‡ Unaffiliated, Quickborn, Germany; § Natural Resources Canada, Canadian Forest Service, Great Lakes Forestry Centre, Sault Ste. Marie, Canada

**Keywords:** Black fungus gnats, *Pinus banksiana*, photoeclector, Boreal zone, new taxa

## Abstract

A new species of black fungus gnat from Canada, *Peyerimhoffia jaschhoforum* **sp. n.**, is presented with a description, illustrations, biotope information and a brief discussion of the placement and concept of the genus *Peyerimhoffia*. *P. jaschhoforum* is characterized by a unique gonostylar structure where the apex is hollowed but not enclosed and contains a mass of mega setae housed within. *P. jaschhoforum* was reared from decomposing jack pine (*Pinus banksiana* Lamb.) deadwood using both *in-situ* and *ex-situ* photoeclectors. We documented three additional specimens originating from Fennoscandia that resemble *P. jaschhoforum* but differ based on a broader tegmen, placement of setigerous papillae behind the tegmen and the fused intercoxal area. Based on this, these specimens are assigned to a new subspecies, *Peyerimhoffia jaschhoforum fennoscandica* **ssp. n.**

## Introduction

The Holarctic genus *Peyerimhoffia* Kieffer, 1903 was defined by Vilkamaa and Hippa (2005), who presented a key and described several new species. Since then, additional closely related species have been placed within *Peyerimhoffia* (Hippa and Vilkamaa 2005, Vilkamaa et al. 2013, Shi et al. 2014, Rudzinski and Baumjohann 2009, Rudzinski and Baumjohann 2012) including species formerly included in the *Corynoptera crassistylata* group *sensu* Menzel and Mohrig (2000). However the concept of the genus in its current state is disputed with authors continuing to include *crassistylata* group species in *Corynoptera* (Menzel et al. 2011, Shin et al. 2013). Until a new analysis of the phylogenetic relationships between representatives is presented, we follow the concept of Vikamaa and Hippa. The following paper presents a new, slightly deviant addition to the genus, *Peyerimhoffia* jaschhoforum sp. n. and with coeval description of a Northern European subspecies, *Peyerimhoffia jaschhoforum fennoscandica* ssp. n.

## Materials and methods

Thirty five specimens of the new species were collected in Ontario, Canada. Nine male specimens were collected from a jack pine (*Picea banksiana*) log (Fig. 1a) on the 22 July 2013 and again on the 6 August 2013 located in a closed canopy jack pine forest (47.572 -82.859) near Chapleau, Ontario, Canada. The log (29 cm ø) was in the early stages of decay (decay class 1 based on Rouvinen et al. 2002), with well intact bark. Six other specimens were collected from a similar log (decay class 1, 22cm ø) in the same forest. Two specimens were collected on the 22 July 2013, 6 August 2013 and 19 August 2013 respectively. All specimens were collected using *in-situ* photoeclectors (Fig. 1b) identical to those described in (Work and Hibbert 2011). Twenty male specimens were also collected from 70 cm log sections taken from a neighboring closed canopy jack pine forest (47.636 -83.243). These specimens were reared from logs in sonotubes between 19 May and 14 September 2013. Eighteen specimens were reared from a log section in advanced stages of decay (decay class 4, 9.5 cm ø). Two additional specimens were reared from separate log sections in advanced stages of decay (decay class 4, 17 cm ø). These specimens were collected from a broader study examining the ecological impacts of intensive biomass harvesting on saproxylic biodiversity. European specimens were collected by Catrin and Mathias Jaschhof in boreal mixed forests during expeditions in Northern Europe. Two specimens were taken with an aspirator and one with a sweep net.

Specimens were sorted using Nikon SMZ800 or Hertel & Reuss STE-5R stereo microscopes and stored in 70% ethanol. Type specimens were selected, dehydrated in 96% ethanol, dissected and slide mounted in Euparal or in Canada Balsam. Specimens were observed under an ISO9001 compound microscope with magnifications of 40×, 100× and 400×.

Specimens were photographed using a MCA-510 USB microscope camera by TUCSEN (Xintu Photonics Co., Ltd.). Between 15–40 images taken at different focal lengths were merged with the aid of the Public Domain Software CombineZP using the method “Weighted Average”. Using GIMP software version 2.8.0., the colour images were converted to greyscale, contrast, and brightness were enhanced and a filter was applied to accentuate the outlines. After manual redrawing of the printed images and a subsequent greyscale scanning at 600 DPI, the final retouch was accomplished again using GIMP. We used scanning electron microscopy (Hitachi S-3400N Scanning Electron Microscope) to characterize the hypopygium. Prior to taking photos, the male gonostyli were dissected from the gonocoxites in 70% alcohol, transferred to 96% ethanol and dried. Gonostyli and gonocoxites were mounted on a single 12.7 mm aluminium specimen stub with epoxy resin and coated with platinum in preparation for secondary electron imaging. All photos were taken at an 11–19 mm working distance from the specimen. Species descriptions were prepared using DELTA (DEscription Language for TAxonomy) (Dallwitz et al. 1999). The following acronyms correspond to the museums and collections where specimens reside: CNC – Canadian National Collection of Insects, Ottawa, Canada; MZHF – Finnish Museum of Natural History (Zoological Museum), University of Helsinki, Helsinki, Finland; SDEI – Senckenberg Deutsches Entomologisches Intitut, Müncheberg, Germany; PWMP – Private Collection of Werner Mohrig, Poseritz, Germany; PKHH – Private collection of Kai Heller, Quickborn, Germany; PRDM – Private collection of Rob Deady, UQÁM, Montréal.

## Taxon treatments

### Peyerimhoffia jaschhoforum

Heller & Deady, 2014 sp. n.

urn:lsid:zoobank.org:act:D0CBBA0F-B610-470F-95B1-5355196693C4

#### Materials

- **Type status:** Holotype. **Occurrence:** catalogNumber: KH8539; recordedBy: Rob Deady & Tim Work; individualCount: 1; sex: male; lifeStage: adult; preparations: slide; **Taxon:** scientificName: Peyerimhoffia jaschhoforum; genus: Peyerimhoffia; specificEpithet: jaschhoforum; scientificNameAuthorship: Heller & Deady, 2014; **Location:** country: Canada; countryCode: CA; stateProvince: Ontario; county: Sudbury; municipality: Chapleau; locality: Superior forest; verbatimElevation: 460 m; decimalLatitude: 47.572; decimalLongitude: -82.859; **Event:** samplingProtocol: photoeclector; eventDate: 08/06/2013; startDayOfYear: 128; endDayOfYear: 219; year: 2013; month: 8; day: 6; habitat: *Pinus banksiana* forest; **Record Level:** institutionCode: CNC
- **Type status:** Paratype. **Occurrence:** recordedBy: Rob Deady & Tim Work; individualCount: 3; sex: male; lifeStage: adult; preparations: slide; **Taxon:** scientificName: Peyerimhoffia jaschhoforum; genus: Peyerimhoffia; specificEpithet: jaschhoforum; scientificNameAuthorship: Heller & Deady, 2014; **Location:** country: Canada; countryCode: CA; stateProvince: Ontario; county: Sudbury; municipality: Chapleau; locality: Superior forest; verbatimElevation: 460 m; decimalLatitude: 47.572; decimalLongitude: -82.859; **Event:** samplingProtocol: photoeclector; eventDate: 08/06/2013; startDayOfYear: 128; endDayOfYear: 219; year: 2013; month: 8; day: 6; habitat: *Pinus banksiana* forest; **Record Level:** institutionCode: CNC
- **Type status:** Paratype. **Occurrence:** recordedBy: Rob Deady & Tim Work; individualCount: 1; sex: male; lifeStage: adult; preparations: slide; **Taxon:** scientificName: Peyerimhoffia jaschhoforum; genus: Peyerimhoffia; specificEpithet: jaschhoforum; scientificNameAuthorship: Heller & Deady, 2014; **Location:** country: Canada; countryCode: CA; stateProvince: Ontario; county: Sudbury; municipality: Chapleau; locality: Superior forest; verbatimElevation: 460 m; decimalLatitude: 47.572; decimalLongitude: -82.859; **Event:** samplingProtocol: photoeclector; eventDate: 08/06/2013; startDayOfYear: 128; endDayOfYear: 219; year: 2013; month: 8; day: 6; habitat: *Pinus banksiana* forest; **Record Level:** institutionCode: PWMP
- **Type status:** Paratype. **Occurrence:** recordedBy: Rob Deady & Tim Work; individualCount: 1; sex: male; lifeStage: adult; preparations: slide; **Taxon:** scientificName: Peyerimhoffia jaschhoforum; genus: Peyerimhoffia; specificEpithet: jaschhoforum; scientificNameAuthorship: Heller & Deady, 2014; **Location:** country: Canada; countryCode: CA; stateProvince: Ontario; county: Sudbury; municipality: Chapleau; locality: Superior forest; verbatimElevation: 460 m; decimalLatitude: 47.572; decimalLongitude: -82.859; **Event:** samplingProtocol: photoeclector; eventDate: 08/06/2013; startDayOfYear: 128; endDayOfYear: 219; year: 2013; month: 8; day: 6; habitat: *Pinus banksiana* forest; **Record Level:** institutionCode: PKHH
- **Type status:** Paratype. **Occurrence:** recordedBy: Rob Deady & Tim Work; individualCount: 9; sex: male; lifeStage: adult; preparations: ethanol; **Taxon:** scientificName: Peyerimhoffia jaschhoforum; genus: Peyerimhoffia; specificEpithet: jaschhoforum; scientificNameAuthorship: Heller & Deady, 2014; **Location:** country: Canada; countryCode: CA; stateProvince: Ontario; county: Sudbury; municipality: Chapleau; locality: Superior forest; verbatimElevation: 460 m; decimalLatitude: 47.572; decimalLongitude: -82.859; **Event:** samplingProtocol: photoeclector; eventDate: 08/06/2013; startDayOfYear: 128; endDayOfYear: 219; year: 2013; month: 8; day: 6; habitat: *Pinus banksiana* forest; **Record Level:** institutionCode: PRDM
- **Type status:** Paratype. **Occurrence:** recordedBy: Tim Work & Rob Deady; individualCount: 20; sex: male; lifeStage: adult; preparations: slide; **Taxon:** scientificName: Peyerimhoffia jaschhoforum; genus: Peyerimhoffia; specificEpithet: jaschhoforum; scientificNameAuthorship: Heller & Deady, 2014; **Location:** country: Canada; countryCode: CA; stateProvince: Ontario; county: Sudbury; municipality: Chapleau; locality: Nimitz; verbatimElevation: 470 m; decimalLatitude: 47.636; decimalLongitude: -83.243; **Event:** samplingProtocol: sonotube; eventDate: 09/14/2013; startDayOfYear: 140; endDayOfYear: 219; year: 2013; month: 9; day: 14; habitat: *Pinus banksiana* forest; **Record Level:** institutionCode: CNC

#### Description

Male. **Head**. Eye bridge 1–2 rows of facets. Antennae unicolour. LW-index of 4^th^ antennal flagellar segment 1.35–1.6; neck 0.25–0.37 × segment width; Transition of basal part to neck pronounced with hairs shorter than segment width; these hairs of normal strength and adjacent. (Fig. 2d). Colour of neck unicolour. Palpi darkened; palpi short; palpomeres 2. First palpomere of normal shape; with 2–4 bristles and only some sparse sensillae. Second palpomere shortly oval and with 3–5 bristles (Fig. 2f). **Thorax**. Colour brown. Notum unicolour. Thoracic setae weak; brown. Posterior pronotum bare. Mesothoracic sclerites bare. *Legs*. Colour yellow-brown. Hind coxae darkened. Hairs on fore coxae bright. Front tibia apically without special structure, however, a comblike structure is visible (Fig. 2c). Front tibial organ bright and unbordered. Tibial setae on hind legs weak, inconspicuous. Tibial spurs of equal length. Claws untoothed. *Wings* (Fig. 4b). Wings slightly darkened; of normal shape. Wing membrane without macrotrichia. Wing venation weak, with faint m-base. M-fork of normal shape. R_1_ inserting clearly before base of m-fork; posterior veins bare; bM bare; r-m bare; bM:r-M 1.46–1.7; st-Cu:bM 0.15–0.4; r_1_:r 0.4–0.5; C:w 0.55–0.66. Halteres dark; of normal length. **Abdomen**. Abdominal setae weak; dorsally brown. Hypopygium (Fig. 2a) concolour with abdomen; 0.7–0.8 × longer than wide. Base of gonocoxites bare; gonocoxites broadly separated; inner margin of gonocoxites broadly extended; inner membrane of hypopygium bare or scarcely setose; elongated setae on valves of hypopygium present. Gonostylus elongate (Figs 2a, b, 3a) 1.7–2 × longer than wide; Inner margin concave; apex tapered. Apical tooth present; as long or longer than subapical megasetae; 1.5–2.5 × longer than broad; strong; with ventral opening containing setae (Figs 2b, 3a, b, c). Awl-like setae absent. Megasetae on inner part of gonostylus present; number of megasetae 3; thick; curved; in one group; Position of lowest megaseta 8–15% from top. Whiplash-hair absent. Tegmen (Fig. 2e) 1–1.3 × longer than broad; equally rounded; normal; Central process absent, setigerous papillae present: apically and centrally located behind tegmen. Field with aedeagal teeth present. Length of ejaculatory apodeme/hypopygium 20–27%; Aeadeagal apical structure absent. **Measurements**. Body size 1.5–1.8 mm. Hind tibia 0.5–0.6 mm. Wing length 1.2–1.6 mm.

#### Diagnosis

*P. jaschhoforum* (Fig. 4a) is instantly recognizable by its long, drawn-out tegmen (Fig. 2a, e) that extends up to the base of the gonostyles. It also has a characteristic set of megasetae-like bristles (3-4) that are bunched together and slotted into the underside/ventral part of the hollow apical tooth which is reminiscent of an upturned canoe (Figs 2b, 3b, c). On one of the paratypes we examined, the ventromesial sclerotization of the gonostylus was ruptured (Fig. 2b). This forced the megasetae-like bristles out from the sheath-like tooth that normally houses the bristles (Fig. 3b, c). When viewed with a fibre-optic lamp or otherwise, these setae may be visible within the tooth giving the illusion of surface topography/texture on the tooth. *P. jaschhoforum* can be separated from most similar looking species by the absence of long specialized setae and megasetae on the inner-sides of the gonostyles.

#### Etymology

*Peyerimhoffia jaschhoforum* is named in honour of Catrin and Mathias Jaschhof in recognition of their work on Sciaroidea and who collected provisional specimens from Northern Europe.

#### Distribution

Boreal zone of Nearctic Region.

#### Ecology

*P. jaschhoforum* appears to be associated with both early and advanced stages of decaying deadwood. In early stages of decomposition larvae most likely reside underneath the bark as interior wood is still intact. The affinity with deadwood likely explains why this species and other *Peyerimhoffia* species tend to be collected at and around ground level close to the soil surface (Vilkamaa and Hippa 2005). *Peyerimhoffia* species also tend to be some of the most minute Sciaridae potentially inferring poor dispersal ability. This suggests that Malaise traps may be relatively inefficient in sampling these species.

#### Taxon discussion

*P. jaschhoforum* appears to be a transitional form between the true *Peyerimhoffia* species such as *P. vagabunda* which have reduced palpi and a practically undifferentiated tibial organ and *Peyerimhoffia* s.l., formerly the *Corynoptera crassistylata* group. In *P. jaschhoforum*, the narrowly elongated gonostyles resembles *Peyerimhoffia* species such as *P. thula*, *P. collina* and *P. semicurvata*. The setigerous papillae behind the tegmen possibly suggests a relation with *P. alpina*, also belonging to the former *Corynoptera crassistylata* group *sensu* Menzel and Mohrig (2000). For these reasons, we placed it in the genus *Peyerimhoffia*.

In the current concept of *Peyerimhoffia* *sensu* Vilkamaa and Hippa (2005), the absence or reduction of long, specialized setae may mislead the observer and suggest *P. jaschhoforum* is not part of *Peyerimhoffia*. However, modification on the mesial side of the gonostyles has been found in other enigmatic species, such as *P. sepei* (Hippa and Vilkamaa 2005). In *P. jaschhoforum*, we record the first reduction in these specialized setae. When compared to *P. alpina*, the sole Nearctic *Peyerimhoffia* species described to date, *P. jaschhoforum* differs in that the intercoxal area is not fused and the tegmen is narrower and longer. However the absence of long specialized setae at the inner side of the gonostyles isolates this species from all other potential congeners. Given the small size and the absence of recognizable characters, additional genetic characters will be helpful to correctly position *P. jaschhoforum* in a phylogeny. The interesting makeup of the gonostylar tooth and associated setae merits larger comparisons across the true *Peyerimhoffia* and *Peyerimhoffia* s.l. using scanning electron microscopy (SEM). It is possible that *Peyerimhoffia* as a genus *sensu* Vilkamaa and Hippa is incorrect. The intermediate characters of *P. jaschhoforum* further suggests that the genus *Peyerimhoffia* may be polyphyletic.

### Peyerimhoffia jaschhoforum fennoscandica

Deady & Heller, 2014 subsp. n.

urn:lsid:zoobank.org:act:E4A1B9B4-A6BF-497C-A3F5-B2F63B09A06A

#### Materials

- **Type status:** Holotype. **Occurrence:** recordedBy: Catrin & Mathias Jaschhof; individualCount: 1; sex: male; lifeStage: adult; preparations: slide; **Taxon:** scientificName: Peyerimhoffia jaschhoforum fennoscandica; genus: Peyerimhoffia; specificEpithet: jaschhoforum; infraspecificEpithet: fennoscandica; scientificNameAuthorship: Deady & Heller, 2014; **Location:** country: Sweden; countryCode: SE; stateProvince: Lapland; municipality: Arjeplog; locality: Lake Sädjavaure; verbatimElevation: 750 m; verbatimLatitude: 66°31'39" N; verbatimLongitude: 16°27'23" E; decimalLatitude: 66.52750; decimalLongitude: 16.45639; **Event:** samplingProtocol: pooter/aspirator; eventDate: 07/07/2004; endDayOfYear: 189; year: 2004; month: 7; day: 7; habitat: subalpine birch forest; **Record Level:** institutionCode: SDEI
- **Type status:** Paratype. **Occurrence:** catalogNumber: KH6552; recordedBy: Mathias Jaschhof; individualCount: 1; sex: male; lifeStage: adult; preparations: slide; **Taxon:** scientificName: Peyerimhoffia jaschhoforum fennoscandica; genus: Peyerimhoffia; specificEpithet: jaschhoforum; infraspecificEpithet: fennoscandica; scientificNameAuthorship: Deady & Heller, 2014; **Location:** country: Finland; countryCode: FI; stateProvince: North Karelia; county: Pielinen Karelia; municipality: Lieksa; locality: Jongunjoki National Park; verbatimElevation: 115 m; verbatimLatitude: 63°27'50" N; verbatimLongitude: 30°06'16" E; decimalLatitude: 63.46389; decimalLongitude: 30.10444; **Event:** samplingProtocol: sweepnetting; eventDate: 07/18/2004; endDayOfYear: 200; year: 2004; month: 7; day: 18; habitat: spruce, pine, birch forest; **Record Level:** institutionCode: PKHH
- **Type status:** Paratype. **Occurrence:** catalogNumber: FI9395; recordedBy: Mathias Jaschhof; individualCount: 1; sex: male; lifeStage: adult; preparations: slide; **Taxon:** scientificName: Peyerimhoffia jaschhoforum fennoscandica; genus: Peyerimhoffia; specificEpithet: jaschhoforum; infraspecificEpithet: fennoscandica; scientificNameAuthorship: Deady & Heller, 2014; **Location:** country: Finland; countryCode: FI; stateProvince: Central Finland; county: Saarijärvi Viitasaari; municipality: Saarijärvi; locality: Pyhä-Häkki National Park; verbatimElevation: 140 m; verbatimLatitude: 63°52'00" N; verbatimLongitude: 25°26'00" E; decimalLatitude: 63.86667; decimalLongitude: 25.43333; **Event:** samplingProtocol: pooter/aspirator; eventDate: 07/03/2004; endDayOfYear: 185; year: 2004; month: 7; day: 3; habitat: spruce, birch, alder, pine forest along stream; **Record Level:** institutionCode: MZHF

#### Description and Diagnosis

**Figure 5.**
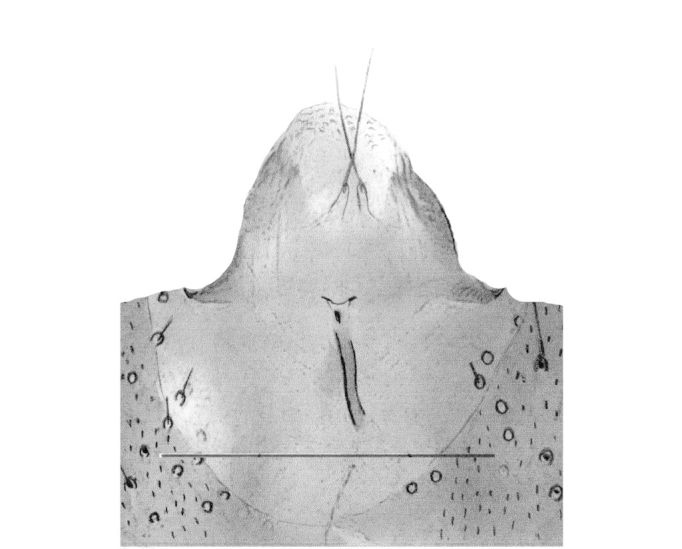
Tegmen of *P. j. fennoscandica* ssp. n. (scale: 0.05 mm).

The main characters are basically the same as in the nominate subspecies described above. Referring mainly to (Fig. 5), *P. j. fennoscandica* differs in the following ways:

- the hypopygium is slightly larger
- the apical tooth is narrower and hooked
- the gonostyles are more tumid
- the intercoxal area is fused and U-shaped
- the first palpomere contains 1–3 bristles
- the tegmen is broader and shorter with darkened lateral edges
- the setigerous papillae are centrally located behind the tegmen when viewing ventrally

#### Etymology

The subspecies was named after the region Fennoscandia where it has been collected.

#### Distribution

Boreal zone of Palaearctic Region.

#### Ecology

The method used to collect specimens of *P. j. fennoscandica* was non-substrate specific (aspirator and sweep-net). It is therefore difficult to comment on its ecology. As it was found in mixed subalpine forest it appears to be forest associated but any deadwood associations are unconfirmed until more substrate specific sampling is carried out.

## Supplementary Material

XML Treatment for Peyerimhoffia jaschhoforum

XML Treatment for Peyerimhoffia jaschhoforum fennoscandica

## Figures and Tables

**Figure 1a.**
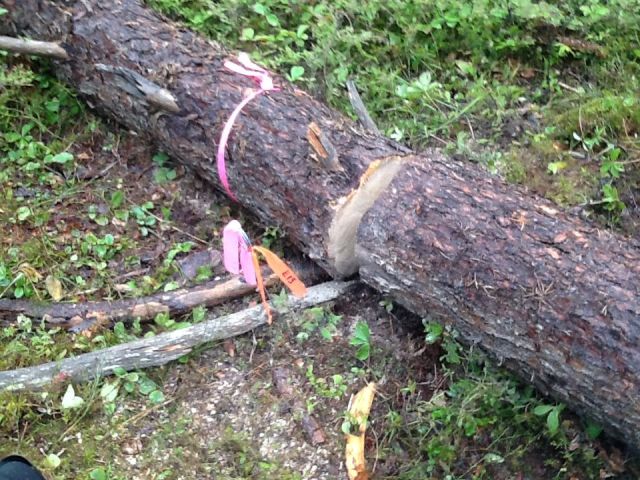
One of the logs at Superior forest where nine specimens including the holotype were found.

**Figure 1b.**
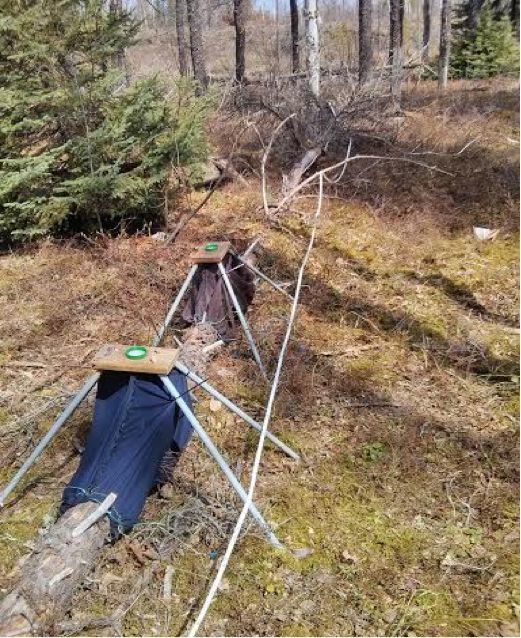
Two *in-situ* photoeclectors primed for use in Superior forest.

**Figure 2a.**
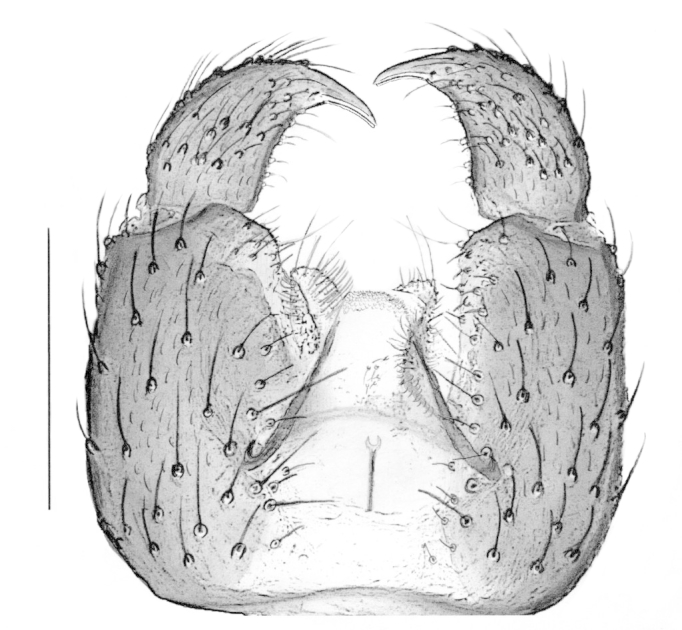
Hypopygium (scale: 0.1 mm).

**Figure 2b.**
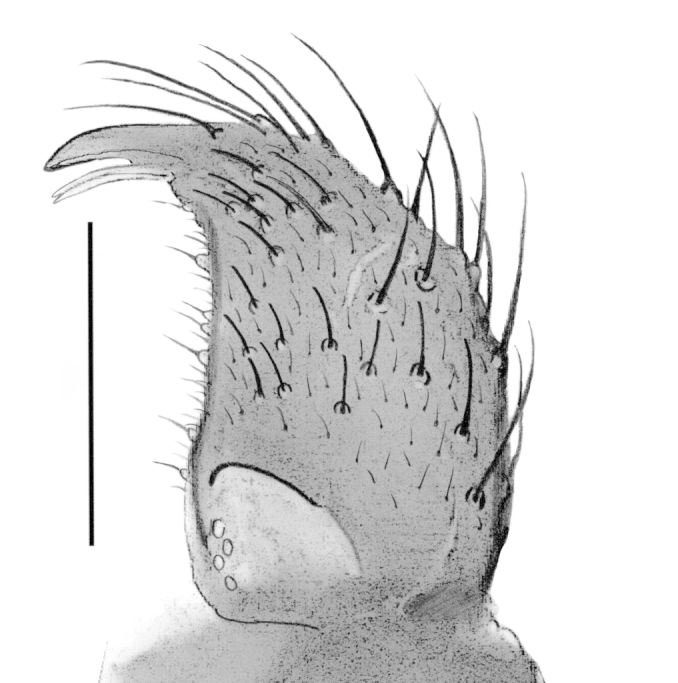
Gonostylus (scale: 0.05 mm).

**Figure 2c.**
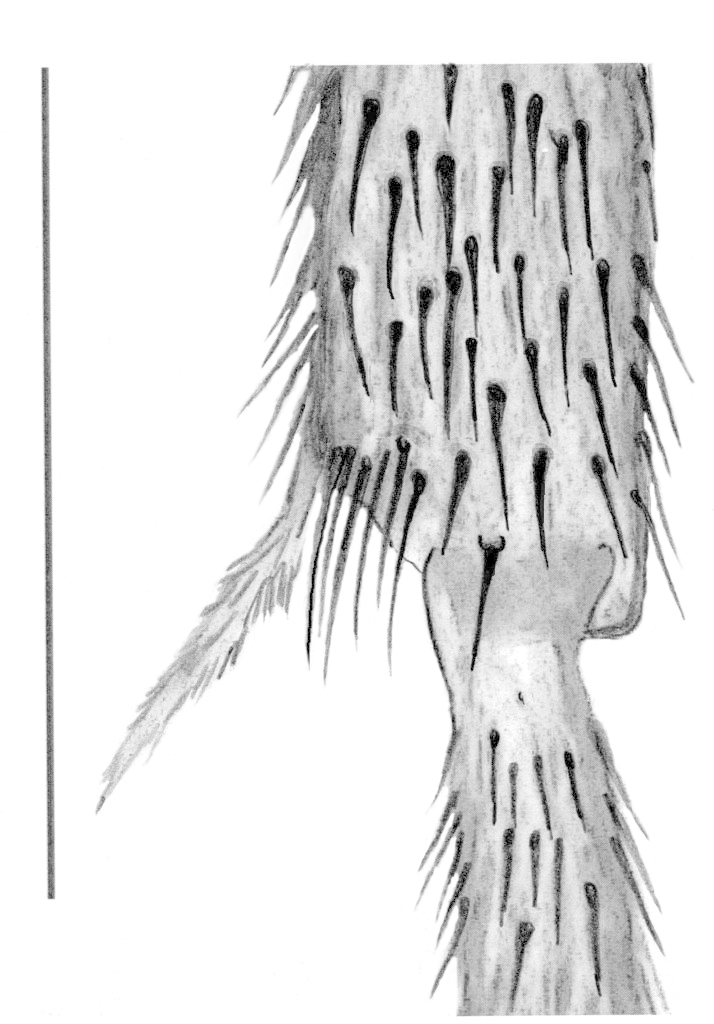
Fore tibial armature (scale: 0.1 mm).

**Figure 2d.**
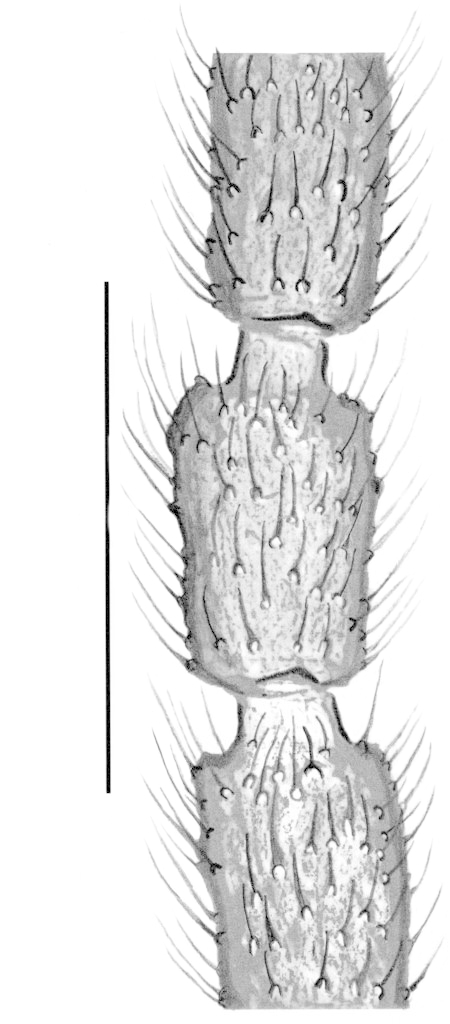
3^rd^, 4^th^ and 5^th^ antennal segments (scale: 0.1 mm).

**Figure 2e.**
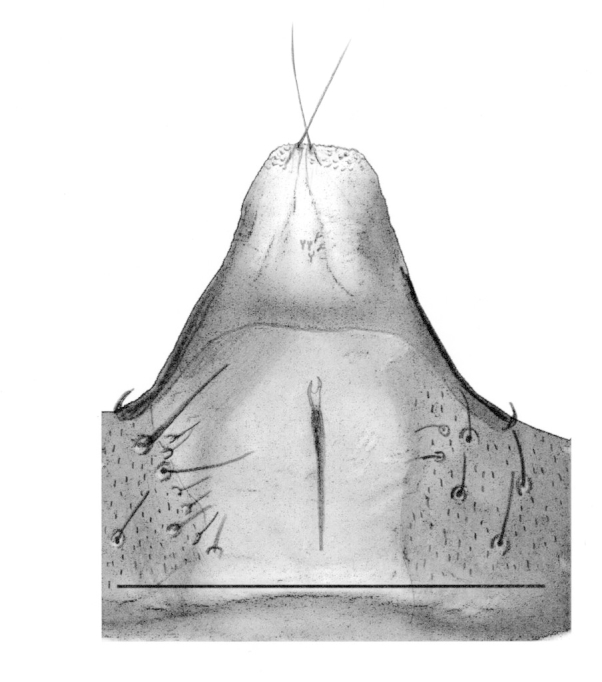
Tegmen and aedeagus (scale: 0.05 mm).

**Figure 2f.**
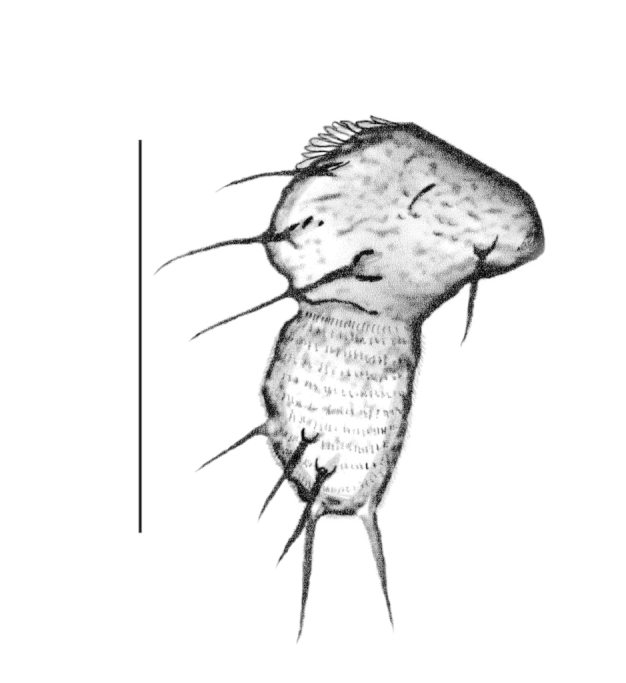
Palpus (scale: 0.05 mm).

**Figure 3a.**
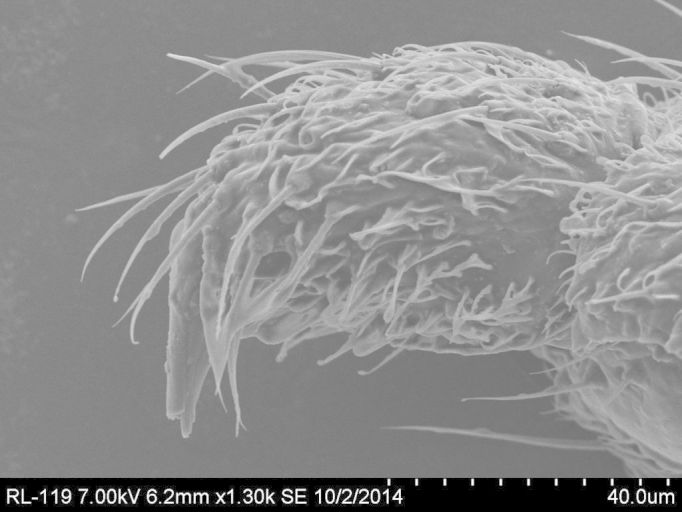
SEM image of male gonostylus (ventral). Magnification 1300× (6.2 mm working distance).

**Figure 3b.**
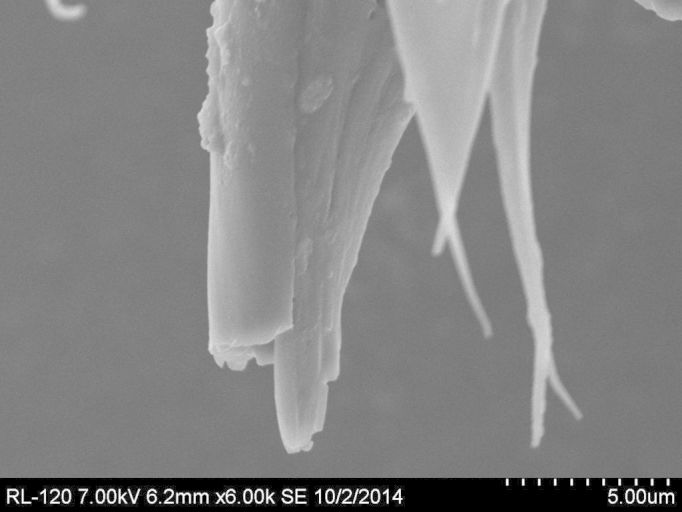
SEM image of broken gonostylar sheath (ventral) revealing the upper surface of a dense mass of megasetae. Magnification 6000× (6.1 mm working distance).

**Figure 3c.**
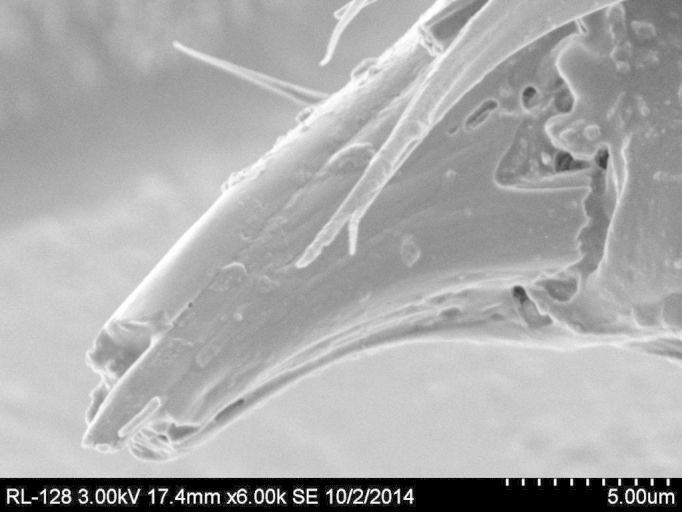
SEM image of gonostylar tooth, rotated 50 degrees and revealing clearly defined edges of sheath-like process covering megasetae. Magnification 6000× (17.4 mm working distance).

**Figure 4a.**
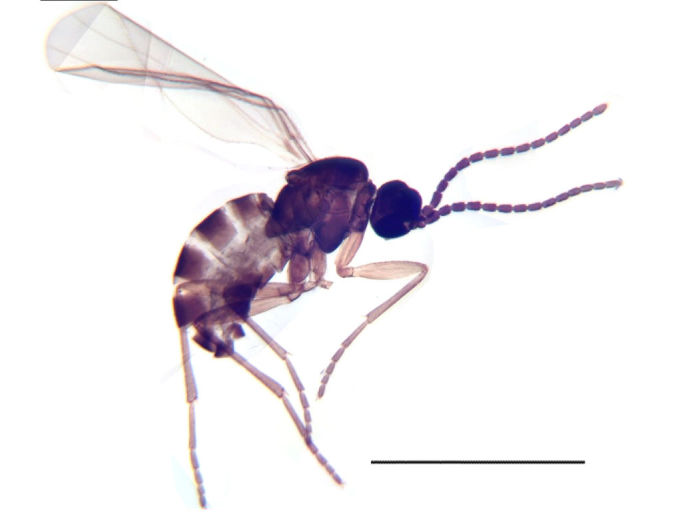
Habitus (scale: 1 mm).

**Figure 4b.**
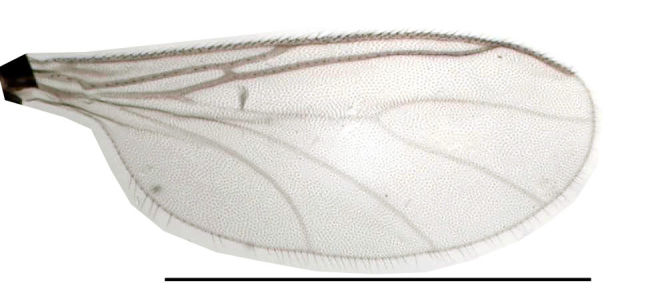
Wing (scale: 1 mm).
